# Salmon (*Salmo salar)* Cooking: Achieving Optimal Quality on Select Nutritional and Microbiological Safety Characteristics for Ready-to-Eat and Stored Products

**DOI:** 10.3390/molecules25235661

**Published:** 2020-12-01

**Authors:** Artur Głuchowski, Ewa Czarniecka-Skubina, Jarosława Rutkowska

**Affiliations:** Department of Food Gastronomy and Food Hygiene, Institute of Human Nutrition Sciences, Warsaw University of Life Sciences (WULS), Str. Nowoursynowska 166, 02-787 Warsaw, Poland; artur_gluchowski@sggw.edu.pl (A.G.); jaroslawa_rutkowska@sggw.edu.pl (J.R.)

**Keywords:** salmon, sous-vide, roasting, steaming, chemical composition, fatty acids, microbiology, cooking loss

## Abstract

This study was performed in order to assess technological characteristics, proximate composition, fatty acids profile, and microbiological safety of sous-vide processed salmon in comparison with steaming and roasting. The cooking loss was lower in the sous-vide method (6.3–9.1%) than in conventional methods (11.6–16.2%). The preparation of salmon using sous-vide was more time- and energy-consuming than steaming. The dry matter content of the salmon fillets was higher in conventionally processed samples than sous-vide due to the evaporation of water, and it was connected with total protein (r = 0.85) and lipid content (r = 0.73). Analysis of the fatty acids profile only revealed significant differences in six fatty acids. All of the heat treatment methods ensured microbiological safety with regard to coagulase-positive *Staphylococcus*, *E. coli*, *Listeria monocytogenes*, and *Salmonella* spp. However, in sous-vide (57 °C, 20 min) and steamed samples after storage Enterobacteriaceae bacteria (<10^4^) was detected. Summing up, high parameters of sous-vide salmon cooking, when considering both technological parameters, nutritional value, and microbiological status should be recommended.

## 1. Introduction

Fish, including salmon (*Salmo salar*), is an essential component of a balanced diet and it provides dietary protein, minerals, vitamins (i.e., A, D, Niacin, and B_12_), and other valuable nutrients, including long-chain (LC) n-3 polyunsaturated fatty acids (PUFA), such as eicosapentaenoic acid (EPA) and docosahexaenoic acid (DHA) [1,2]. Health benefits, such as lower risk of coronary heart disease and stroke, are associated with consumption of various fish species, though the benefits depend on harvesting and cultivation practices as well as culinary processing practices and portion sizes [3]. Fish is usually consumed after being subjected to various heat treatment methods: grilling, roasting, frying, or steaming, as well as, more recently, sous-vide. Cooking processes, including cooking temperature, amount of time cooked, and the use of water affect not only nutritional value, but also the sensory and microbiological quality of the fish. A review of literature data revealed the effect of thermal processing on proximate composition and the fatty acids profile of salmon [4,5,6,7]. However, little research has focused on the sous-vide method of cooking fish [8,9].

In the USA, seafood is more likely than other foods to be purchased at restaurants and other foodservice outlets. Additionally, sixty-five percent of American consumer expenditures for seafood were at restaurants [10]. Salmon is the most popular species in retail sales in the USA, but also in Germany and France [11].

The sous-vide method differs from conventional heat treatment by the use of vacuum sealing raw materials in thermostable pouches and cooking them in controlled conditions, usually at a temperature of 65–95 °C [12].

According to the literature, the parameters for the heat treatment of fish are not always sufficient for achieving satisfactory microbiological quality. An equivalent of 70 °C/2 min guaranteed a reduction of *Listeria monocytogenes*, but, in some instances [13,14,15], the fish was cooked at a temperature below 60 °C, and not long enough to reach this equivalent. Still, one recent study [16] demonstrated that a processing temperature range of 40–50 °C still secures the inhibitory growth effect on *Listeria* spp.

The intensity of heat treatments and cooling processes, as well as storage temperature and its control, affect the quality and shelf life of sous-vide products. Previous research has described the influence of these factors on the quality (including the nutritional value, and sensory and microbiological quality) of different fish species [17,18,19]. Previous studies [9,16,20,21,22,23,24] discuss salmon prepared by sous-vide, but in a different context than our study. Our recent results [24] revealed that low parameter sous-vide processes resulted in products with similar characteristics to raw salmon, while higher parameters achieve a high intensity cooked fish odor and flavor without significant deterioration in texture.

Fish and seafood waste, along with discarded cereal, vegetables, and fruits, constitute the largest portions of economic loss in the food service industry [25]. The results of Bilska et al. [26] revealed that unsold food is usually refrigerated until the next day or trashed, and forty percent of establishments studied disposed of expired food and food waste in a dumpster. The sous-vide method is usually used in order to extend the shelf life of food products and, for several years, it has been used in catering to reduce food waste that is caused by overproduction. Most of the research [16,27] on sous-vide salmon concerns storage conditions and raw product quality, including microbiological quality, after storage. In gastronomy, pre-prepared dishes could be used in a subsequent day’s food service or delivered to customer’s home for later use. Some recent studies [8,9,24] have been dedicated to comparing the sous-vide method to other cooking methods in salmon processing, but no studies took into consideration the energy consumption of the sous-vide process. Sous-vide as a cooking method can be a good solution for one-person households, which are increasing in Europe. Moreover, it could appeal to many people looking for natural, convenient food products without the use of food additives.

The aim of this study was to assess the technological characteristics (cooking loss, time, and energy consumption), proximate composition and fatty acids profile, as well as microbiological quality of the sous-vide salmon process when compared to conventional methods (steaming and roasting) and to show the advantages and disadvantages of each method. The sous-vide cooking method is used to reduce food waste and overproduction, but it is important that these benefits are also considered alongside energy use and waste. For this reason, we also estimated electric energy consumption.

## 2. Results

### 2.1. Technological Characteristics of Chosen Cooking Methods

The cooking losses of salmon fillets using conventional methods (11.6–16.2%) were higher than both sous-vide samples: SV_57_ and SV_63_ (6.3 and 9.1%), as in Table 1. The total process duration of sous-vide method at both temperatures (57, 63 °C) was higher than other methods. Despite this, these methods were not associated with greater energy consumption when compared with roasting. Steaming consumed less energy than sous-vide methods (Table 1).

Salmon that was processed with the sous-vide method at 57 °C (SV_57_) had the least changed pH when compared with the raw samples (Table 1). Salmon that was prepared with other methods (SV_63_, SP_100_, R_180_) differed significantly in pH from raw samples (*p* ≤ 0.05).

### 2.2. Proximate Composition and Fatty Acids Profile in Salmon after Cooking

The dry matter content of the salmon fillets was higher after traditional processing methods (39.3–41.1%) than in either sous-vide methods (37.2–38.8%) and it was related with total protein (r = 0.85, *p* ≤ 0.05) and lipid content (r = 0.73, *p* ≤ 0.05). Slightly processed salmon fillets (SV_57_—57 °C, 20 min) had a similar proximate composition (protein and fat content) with raw material (although significantly different). A thirty-five percent increase in lipid content was assayed in the case of SV_57_ samples, 80.9 % in SV_63_ samples, while, in the conventionally heat-treated samples, it ranged between 101.5–111.8 % (Table 2). The protein content also increased—in the range of 14.9–21.9%—but the results did not differ between the cooking methods.

Twenty-eight FAs were identified in fat that was extracted from both raw and cooked salmon. However, only the content of six of them differed significantly depending on the method used. The FAs composition of raw salmon fillets was characterized by the highest content of monounsaturated fatty acids (MUFA) (46.6 g/100 g) and a substantial amount of polyunsaturated fatty acids (PUFA) (32.1 g/100 g). The smallest group of fatty acids were saturated SFA (15.9 g/100 g), Table 2.

Among MUFA, whose relative content after heat treatment increased insignificantly, oleic acid had the largest share in the group (C18:1 9c). Palmitic acid (C16:0) dominated in the SFA group. Linoleic acid (C18:2) was the largest in the PUFA group.

Heat treatment did not significantly (*p* > 0.05) affect the fatty acid profile of the salmon filet in the content of SFA and MUFA. However, processing with the sous-vide method SV_57_ (57 °C, 20 min) resulted in the least amount of change. This sample had a significantly (*p* ≤ 0.05) higher content of PUFA when compared with salmon samples that were cooked in sous-vide SV_63_ and steaming, but they did not differ from roasted salmon. The ratio of n-6/n-3 FA was similar in raw and heat-treated samples (Table 2).

On the other hand, the sous-vide salmon (SV_63_) had a similar fatty acid profile with samples that were prepared conventionally. Sous-vide salmon fillets (SV_57_) had significantly (*p* ≤ 0.05) higher content of n-3 PUFA (mainly DHA, EPA) compared with other samples. Three methods (SV_63_, R_180_, SP_100_) caused a decrease in DHA content from 14.3 to 17.2%. Steamed salmon had a significantly (*p* ≤ 0.05) lower content of C20:4 arachidonic acid (0.319 g/100 g FAs), while roasted had higher (0.609 g/100 g FAs) than other samples. However, the content of DHA in a sous-vide sample that was cooked at 57 °C did not change.

A calculation of obtained data of EPA and DHA content per 100 g of ready-to-eat salmon revealed that steaming and roasting resulted in their higher content of EPA and DHA than those that were prepared using sous-vide. It is linked with the highest fat content in conventionally cooked samples (Table 3).

### 2.3. Microbiological Quality of Prepared and Cold Storage Salmon

**Figure 1:**
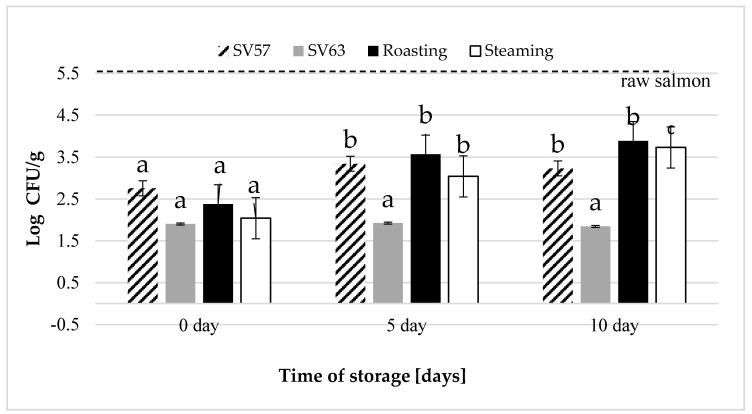
Total Viable Count in salmon processed with various heat treatment methods: a, b, c—mean values that are marked by different letters between time of storage, differ significantly at *p* ≤ 0.05.

Raw salmon was characterized by satisfactory microbiological quality (Figure 1). The initial (0 day) TVC load of a raw fillet was 5.2 log10 CFU/g, while the yeast and mold counts were 2.7 log10 CFU/g, and *Enterobacteriaceae*, 4.7 log10 CFU/g. All heat treatments significantly (*p* ≤ 0.05) influenced the microbial reduction. The count of total mesophilic aerobic bacteria (30 °C /48 h) may be considered as satisfactory (<5 log10 CFU/g) for cooked fish category according to the Expert Panel on Microbiological Safety of Food guidelines [28].

Pathogens: coagulase-positive *Staphylococcus*, *E. coli*, *Listeria monocytogenes*, and *Salmonella* spp. were not detected in any raw or cooked samples. All of the applied heat treatment methods (sous-vide SV_57_, SV_63_, roasting R_180_, and steaming SP_100_) successfully reduced the yeast and mold counts.

*Enterobacteriaceae* (Eb) was not detected in salmon that were processed by sous-vide (SV_63_) or roasting. While *Enterobacteriaceae* was in salmon processed by the sous-vide (SV_57_) and steaming after cold storage for five and 10 days, Eb still did not exceed the unsatisfactory quality limits (>4 log 10 CFU/g) that were established by the Health Protection Agency [29]. In sous-vide samples SV_57_ stored under controlled conditions (2 °C) for five and 10 days, an Eb count was detected (2.2 log10 CFU/g and 2.3 log10 CFU/g, respectively). In steamed samples that reached core temperatures of 70 °C, the count was slightly higher (2.3 log10 CFU/g) at five days and (3.0 log10 CFU/g) at 10 days of cold storage.

The microbiological quality of salmon processed with the slightly increased sous-vide equipment parameters (57 °C, 20 min) does not guarantee full food safety, whereas a higher process parameter (63 °C, 80 min) does.

## 3. Discussion

### 3.1. Technological Characteristics of Processed Salmon

In this study, cooking losses of the sous-vide method increased alongside increased time-temperature combination (from 57 °C for 20 min to 63 °C for 80 min). They were also higher than steaming and roasting processes (SP_100_ and R_180_). These findings were in accordance with the results of model research on salmon [30,31] and sous-vide processed seafood [32]. Husein et al. [8] have reported that a sous-vide salmon fillet had higher moisture than a boiled one. Other authors confirmed a higher cooking loss of salmon baked in foil than steamed [6,7]. Moisture loss results from heat-induced protein denaturation and aggregation, according to Ovissipour, Rasco, Tang & Sablani [31]. Myofibrillar proteins are mainly responsible for the decrease in weight during thermal processing. The largest weight loss occurs at a temperature range of 50–60 °C. The process is connected with myosin (35 °C) and actin (58 °C) denaturation, in which the length of sarcomeres also decreases, and collagen start denatures. At a temperature of 60 °C, the space between individual fibers is closed and the shrinkage of myofibrils begins. Further heating leads to the shrinkage of fibers and a decrease in their ability to hold and bind water in the meat [33,34]. The results of other authors confirmed the increase in the pH value, along with the increase of the cooking parameter intensity by this study [35].

Energy consumption per one serving of salmon prepared with the sous-vide method was about 5–6 times higher than during steaming and it increased along with the increase in time-temperature parameters. The energy consumption of the sous-vide method results primarily from the need to preheat the water bath, and low-temperature cooking itself accounts for just 5–10% of the total value. Although roasting lasted only 23 min, it was characterized by 8–20 times higher energy consumption than sous-vide cooking, which lasted 126–145 min. The current findings support the results of our previous papers on sous-vide processed poultry. Despite the protection of nutrition value, the sous-vide heat treatment method is more suitable for foodservice than home use, because of its high energy consumption [36]. The preparation of many batches, as is common in foodservice settings, would recompense the energy consumption costs of preheating. The use of free warm water sources, such as geothermal springs, or the use of solar panels for electricity to heat product might also counter the high energy costs that are associated with the sous-vide method.

### 3.2. Proximate Composition and Fatty Acids Profile in Salmon

Our results support the view that the protein and lipid content are related to the dry matter content of fish samples. This is consistent with findings from other studies of protein content [5,8,37], as well as toward the lipid content [4]. All this indicate an inverse relationship between moisture content and other macronutrients. The cooking loss is mainly contained water (>85%), and the remaining parts are lipids, collagen or gelatin, muscle fragments, and aggregated sarcoplasmic proteins [30]. Hence, there is no linear relationship between cooking loss and fat and protein contents. Moreover, the bound lipids were released as free lipids during the thermal treatment, which makes them easier to extract [4].

Heat treatment only slightly affected the fatty acid profile of the salmon fillets, and the least changed was sous-vide treated sample (SV_57_). The profile of FA of lipid extracted from raw salmon was similar to those in previous studies [4,38]. Our study revealed that heat treatment slightly changed the FA profile of salmon fillets. Low parameters temperature and vacuum in sous vide SV_57_ seem to protect FAs belonging to n-3 (EPA, DHA) and n-6 (AA arachidonic acid) from PUFA family. Opposite conclusions were reached by Nieva-Echevarría et al. [39], who showed that steaming and the sous-vide method similarly affects the profile of FAs in European bass (*Dicentrarchus labrax*). However, extraordinarily high parameters of the sous-vide method (85 °C, 20 min) were used in those studies. Therefore, the literature recommends steam cooking as a faster, cheaper, and more environmentally friendly method. In the research of Larsen, Quek & Eyres [4], no differences in FAs content in poached, steamed, microwaved, oven-baked, pan-fried salmon samples, were observed. Deep-fried salmon had significantly higher linoleic acid content than raw salmon, which is an increase that is derived from the frying medium. In a study conducted by Orlando et al. [9], no significant differences in the total fatty acid profile between salmon samples (conventional—180 °C for 20 min; sous-vide—65 °C for 20 min; sous-vide—until 60 °C in the core) were observed. Husein et al. [8] similarly reported that different cooking methods did not alter the nutritional profile of the raw fish. There were no significant differences in the fatty acid composition between sous-vide and boiled salmon. Bastías et al. [5] suggests that there were no significant differences of PUFAs between heat-treated salmon samples and raw ones. A higher content of DHA in canned and steamed salmon was revealed.

Similar to our results, Şengör, Alakavuk & Tosun [23] suggested that heat treatments affect the FA profile. They found that grilling and oven baking lead to a higher content of EPA and DHA in salmon. According to Rasińska, Rutkowska, Czarniecka-Skubina & Tambor [40], sous-vide and boiling were more beneficial methods than roasting for preserving PUFA content in rabbit meat.

### 3.3. Microbiological Quality

The microbiological quality of the salmon that was prepared by the sous-vide method was satisfactory. However, in SV_57_ and in steamed salmon, on the 5th and 10th day of storage, an increase in *Enterobacteriaceae* count (Eb) was found. This indicated the inefficiency of lower than recommended heat treatment parameters that are commonly used.

Similar results were obtained by Jørgensen et al. [15], who found that a high percentage (45%) of sous-vide prepared fish was unsatisfactory or of borderline microbiological quality exceeding the limits of *E. coli* and *Enterobacteriaceae*. Similarly, Picouet, Cofan-Carbo, Vilaseca, Ballbè & Castells [41] reported an increase of the total viable count (TVC) and *Enterobacteriaceae* count after sous-vide processing of salmon (50 °C, 20 min). Abel et al. [16] demonstrated that a low temperature (40 or 50 °C) process still allows for obtaining an inhibitory effect on *Listeria* spp.

Mild process parameters should be combined with another heat treatment method. In the report of Li et al. [14], 20 min heating in a water bath combined with 45 s searing turned out to be insufficient to reach the recommended internal temperature of 70 °C.

In our study, using the sous-vide at 63 °C for 20 min allowed for reaching high microbiological quality of salmon fillets. While using different parameters, it is possible to store sous-vide prepared fish for up to 40 days. The sous-vide process ensured complete inactivation of Eb in salmon when the authors used the following parameters: 65 °C (10 min), 90 °C (5 and 10 min), and stored up to 45 days [20]; 80 °C (43 min) for 25 days [21]; and, 85 °C (7–28 min, c.a. 62–65 °C in core) for 30 days. However, coagulase negative *Staphylococci* on the 30th day of storage were detected [42]. Salmon that were processed by sous-vide at 90 °C (10 min) induced significantly greater reduction of mesophilic, psychrophilic, *Micrococcaceae,* and anaerobic count than those prepared traditionally (*p* ≤ 0.05) [12]. The exception was *Enterobacteriacea* count, which did not differ statistically.

## 4. Material and Methods

### 4.1. Material

Experimental material—whole side fillets of farmed Atlantic salmon (Salmo salar) of similar size (4–4.5 kg) and sourced from the same breeding. Fishes were supplied by a direct distributor (Fiord S.A., Poland) in ice packed boxes and then stored at 3 ± 1 °C. Only the middle part of the fillet was used for testing due to the different fat distributions throughout the salmon body [9,43]. Study samples of fillets were all the same sizes and thickness. Fillets were trimmed, portioned, and calibrated to a height of 25 ± 2 mm. One serving portion of the salmon fillet for all cooking methods was 276 ± 21 g.

### 4.2. Heat Treatment Methods

The samples of salmon were prepared while using three heat treatments commonly used in catering: roasting, steaming, and sous-vide.

Sous-vide (SV_57_, SV_63_)—the fish samples were packaged in thermostable polyethylene-polyamide pouches (Hendi, Poland) in a chamber-type vacuum packaging machine (Stalgast, Warszawa, Poland). Subsequently, heat treatment in a sous-vide water bath (Hendi, Poland) was performed. The parameters (57 °C, 20 min and 63 °C, 80 min) of heat treatment were chosen based on literature and during a panel discussion in a preliminary research. The details were presented in Głuchowski, Czarniecka-Skubina, Wasiak-Zys & Nowak [24].

Steaming (SP_100_)—fillets were cooked on a perforated insert in a steamer pot filled with boiling water (100 °C), heated by an induction cooker with 400 W (Stalgast, Warszawa, Poland). The process was performed up to 70 °C in core of fillet (c.a. 16 min).

Roasting (R_180_)—the salmon fillets were packed in an aluminum papillote and roasted in a convection-steam oven in 180 °C (RedFox KE—423 RM GASTRO s.r.o., Praha, Czech Republic). The process was performed up to 70 °C in core of fillet (c.a. 23 min).

Every cooking variant was three replicates (a total of nine fillets).

The final temperature of steaming and roasting was determined by inserting a needle thermocouple (MM 2000, TM Electronics Ltd., Worthing, England) into the approximate geometric center (core of fillet) of each sample.

### 4.3. Technological Characteristics

The cooking loss was calculated, as the percent weight difference between raw and cooked salmon samples relative to the weight of raw samples, in accordance with the following equation:

(1) $$\mathrm{Cooking}\;\mathrm{loss}\;\left(\%\right)=\frac{\mathrm{raw}\;\mathrm{fillet}\;\mathrm{weight}\;\left(g\right)-\mathrm{cooked}\;\mathrm{fillet}\;\mathrm{weight}\;\left(g\right)}{\mathrm{raw}\;\mathrm{fillet}\;\mathrm{weight}\;\left(g\right)}\times \;100$$

Cooking loss was measured in 12 replications.

The electric energy consumption of producing one serving was determined with an energy-monitoring socket (Energy Check 3000, Voltcraft^®^, CEI Conrad International (HK) Ltd., Hong Kong, China). The total energy consumption was calculated by summing up the energy consumption for the cooking process and preheating the devices to the set temperature, while in the sous-vide method an energy consumption of vacuum packaging was also added. The electric energy consumption was measured in three replications for each method.

The total process duration was measured while using a stopwatch and included the time for fillet preparation, time for heating the device to set temperature, the time of heat treatment process, and the vacuum packaging time in the sous-vide method.

The pH of salmon fillets was measured before and after heat treatment while using a WTW 340i pH meter (Wissenschaftlich-Technische Werkstatten GmbH, Weilheim, Germany) with an electrode (SenTix^®^ SP Number 103645, Wissenschaftlich-Technische Werkstatten GmbH, Weilheim, Germany) for direct penetration measurements in meat. The pH value was measured in three replications.

### 4.4. Proximate Composition and Fatty Acids Profile

The determination of dry matter content was carried out by drying samples in an oven at 105 °C to constant weight. The total protein content was measured by the Kjeldahl method [44].

The fat content was evaluated by the Soxhlet method, in agreement with PN-ISO 1444:2000 [45], while using ether extraction.

#### Fatty Acids Composition

Methyl esters of FAs (FAMEs) were prepared by the transmethylation of fat samples using 5 M KOH and methanol as a catalyst. Fatty Acid composition as FAME was analyzed while using an Agilent 7890A (Palo Alto, CA, USA) gas chromatograph that was equipped with a flame ionization detector (GC-FID), a split/splitless injector, and capillary column Restek-2330 (105 m × 0.25 mm I.D. 0.2 µm df; Restek Corp., Santa Clara, CA, USA). Helium was the carrier gas at a flow rate 0.9 mL/min. The parameters of GC analysis: injection of 1 µL, the split ratio 1:50, FID temperature was set at 250 °C. Oven temperature program was set from 100 °C, at the rate of 3 °C/min up to 210 °C. The peaks were identified by comparison with Supelco 37 No.47885-U and PUFA-3 standards No.47085 (Sigma–Aldrich, Poznań, Poland). The FAs in g/100 g total lipids were quantified in relation to internal standard (C23:0), which was added before transesterification to lipid samples [46].

### 4.5. Microbiological Analysis

The microbiological quality assessment was carried out in raw salmon (before storage and cooking), after the thermal heating, and after the storage of cooked fish under controlled conditions (2 °C) for five and 10 days. This storage time was used to compare conventional methods to sous-vide, because classical dishes are only stored for a few hours. The following microbiological assays in triplicates were performed: total viable aerobic count (TVC) [47]; yeast and mold count [48]; coagulase-positive *Staphylococci counts* [49]; beta-glucuronidase-positive *E. coli* count [50]; *Enterobacteriaceae* count [51]; *Listeria monocytogenes* count [52]; and, detection of *Salmonella* spp. [53].

### 4.6. Statistical Analysis

A significant difference between proximate composition, FA content, and TVC was assayed while using ANOVA with Fisher’s Least Significant Difference (LSD) post hoc test. A coefficient of correlation according to Pearson calculation was also computed. A significance level of *p* ≤ 0.05 was used (STATISTICA software version 13.1 PL, StatSoft, Poland).

## 5. Conclusions

The preparation of salmon while using the sous-vide method is time and energy-consuming when compared with steaming. However, in terms of cooking loss, it was more beneficial than roasting.

The quality of sous-vide salmon depends on the time-temperature combination applied. The use of mild parameters of sous-vide (57 °C, 20 min) resulted in lower cooking loss and similar nutritional values as raw samples. However, due to undesirable microbiological quality, sous-vide treatment is not suitable for storage and restitution, because of the presence of *Enterobacteriaceae*. Although no microbiological limits have been exceeded, it seems that this method should be used with extreme caution when using low temperature and short duration cooking.

Salmon processed at 63 °C for 80 min had similar a proximate composition and microbiological quality to steamed and roasted samples. However, it was characterized by lower cooking loss and distinguished by the best microbiological quality after 10 days of storage.

All of the studied heat treatments only slightly influenced the FA profile of salmon and did not detract from it being a valuable source of EPA and DHA. These results provide valuable data for food composition tables and indicate that sous-vide processed salmon have similar nutritional value as raw fish. Mild parameters of sous-vide only slightly influenced the proximate composition and fatty acid profile, whereas, in higher parameters and conventional heat treatment methods, it had a significant effect. When considering both technological parameters, nutritional value, and microbiological safety, as well as sensory quality that is estimated in the results of previous study higher parameters of sous-vide in salmon processing should be recommended in catering.

## Figures and Tables

**Table 1:** Technological quality parameters of salmon processed with various heat treatment methods.

Parameter	Raw	$\mathrm{Heat}\;\mathrm{Treatment}\;\mathrm{Method}\;\bar{x}\;\pm \;\mathrm{SE}$			
		Sous-Vide Method		Roasting (R_180_, 23 min)	Steaming (SP_100_, 16 min)
		57 °C, 20 min (SV_57_)	63 °C, 80 min (SV_63_)		
Total process duration (min)	-	126 ± 0.0	145 ± 0.0	29 ± 1.3	48 ± 0.8
Energy consumption (kWh)	-	0.440 ± 0.02 (0.022) *	0.531 ± 0.01 (0.059) *	0.790 ± 0.01 (0.454) *	0.083 ± 0.01 (0.025) *
Cooking loss (%)	-	6.3 ^a^ ± 0.6	9.1 ^a,b^ ± 0.5	16.2 ^c^ ± 1.6	11.6 ^b^ ± 1.1
pH	6.12 ^a^ ± 0.02	6.30 ^b^ ± 0.01	6.34 ^b,c^ ± 0.01	6.32 ^b^ ±0.03	6.39 ^c^ ± 0.02

* Values in brackets mean average energy consumption of the heat treatment process only. In the case of sous-vide the vacuum packaging is included as well; ^a^, ^b^, ^c^—mean values marked by different letters in rows (between cooking methods), differ significantly at *p* ≤ 0.05.

**Table 2:** Proximate composition and fatty acid content of fat extracted from raw and cooked salmon.

Parameter	Raw	$\mathrm{Heat}\;\mathrm{Treatment}\;\mathrm{Method}\;(\bar{x}\;\pm \;\mathrm{SE})$			
		Sous-Vide Method		Roasting (R_180_, 23 min)	Steaming (SP_100_, 16 min)
		57 °C, 20 min (SV_57_)	63 °C, 80 min (SV_63_)		
Dry matter (%)	37.10 ^a^ ± 0.12	37.23 ^a^ ± 0.15	38.80 ^b^ ± 0.17	41.10 ^c^ ± 0.12	39.30 ^b^ ± 0.12
Protein (%)	20.10 ^a^ ± 0.70	23.10 ^b^ ± 0.80	23.70 ^b^ ± 0.82	24.10 ^b^ ± 0.83	24.50 ^b^ ± 0.85
Lipids (%)	6.79 ^a^ ± 0.24	9.20 ^b^ ± 0.32	12.30 ^c^ ± 0.43	14.40 ^e^ ± 0.50	13.70 ^d^ ± 0.47
Fatty acid profile * (g FA/100 g fat)					
SFA	15.90 ^a^ ± 0.01	15.70 ^a^ ± 0.03	15.70 ^a^ ± 0.02	15.60 ^a^ ± 0.06	15.40 ^a^ ± 0.02
C14:0	2.04 ^a^ ± 0.01	1.97 ^a^ ± 0.02	2.06 ^a^ ± 0.01	2.04 ^a^ ± 0.01	2.05 ^a^ ± 0.01
C15:0	0.19 ^a^ ± 0.00	0.19 ^a^ ± 0.02	0.18 ^a^ ± 0.01	0.19 ^a^ ± 0.01	0.19 ^a^ ± 0.03
C16:0	9.38 ^a^ ± 0.01	9.25 ^a^ ± 0.00	9.29 ^a^ ± 0.02	9.18 ^a^ ± 0.00	9.30 ^a^ ± 0.02
C17:0	0.11 ^a^ ± 0.01	0.11 ^a^ ± 0.00	0.11 ^a^ ± 0.02	0.11 ^a^ ± 0.03	0.11 ^a^ ± 0.01
C18:0	2.82 ^a^ ± 0.02	2.83 ^a^ ± 0.08	2.75 ^a^ ± 0.02	2.74 ^a^ ± 0.01	2.77 ^a^ ± 0.05
C20:0	0.44 ^a^ ± 0.02	0.43 ^a^ ± 0.02	0.45 ^a^ ± 0.04	0.45 ^a^ ± 0.02	0.44 ^a^ ± 0.00
C21:0	0.60 ^a^ ± 0.02	0.60 ^a^ ± 0.00	0.61 ^a^ ± 0.01	0.61 ^a^ ± 0.01	0.32 ^b^ ± 0.02
C22:0	0.20 ^a^ ± 0.01	0.19 ^a^ ± 0.00	0.18 ^a^ ± 0.00	0.18 ^a^ ± 0.00	0.19 ^a^ ± 0.00
C24:0	0.08 ^a^ ± 0.03	0.07 ^a^ ± 0.01	0.06 ^a^ ± 0.04	0.07 ^a^ ± 0.01	0.07 ^a^ ± 0.02
MUFA	46.60 ^a^ ± 0.02	46.50 ^a^ ± 0.06	47.40 ^a^ ± 0.08	47.20 ^a^ ± 0.04	47.30 ^a^ ± 0.02
C16:1 n-7	2.27 ^a^ ± 0.00	2.30 ^a^ ± 0.05	2.33 ^a^ ± 0.00	2.36 ^a^ ± 0.06	2.38 ^a^ ± 0.06
C17:1 (cis-10)	0.14 ^a^ ± 0.14	0.30 ^b^ ± 0.02	0.32 ^b^ ± 0.01	0.34 ^b^ ± 0.02	0.32 ^b^ ± 0.04
C18:1 n-9	35.40 ^a^ ± 0.01	35.10 ^a^ ± 0.09	35.80 ^a^ ± 0.17	35.50 ^a^ ± 0.03	35.70 ^a^ ± 0.08
C18:1 n-7	3.30 ^a^ ± 0.06	3.30 ^a^ ± 0.01	3.31 ^a^ ± 0.01	3.30 ^a^ ± 0.00	3.30 ^a^ ± 0.00
C20:1 n-9	4.28 ^a^ ± 0.01	4.36 ^a^ ± 0.02	4.47 ^a^ ± 0.10	4.45 ^a^ ± 0.01	4.44 ^a^ ± 0.01
C22:1 n-9	0.77 ^a^ ± 0.00	0.77 ^a^ ± 0.01	0.77 ^a^ ± 0.00	0.77 ^a^ ± 0.00	0.77 ^a^ ± 0.00
C24:1 n-9	0.45 ^c^ ± 0.02	0.43 ^b^ ± 0.01	0.38 ^a^ ± 0.01	0.42 ^b^ ± 0.00	0.40 ^b^ ± 0.01
PUFA	32.10 ^b^ ± 0.00	32.40 ^b^ ± 0.02	31.50 ^a^ ± 0.05	31.70 ^a,b^ ± 0.07	31.50 ^a^ ± 0.01
C18:2 n-6 (LA)	14.10 ^a^ ± 0.00	14.20 ^a^ ± 0.00	14.10 ^a^ ± 0.03	14.10 ^a^ ± 0.05	14.00 ^a^ ± 0.01
C18:3 n-6 (GLA)	0.08 ^a^ ± 0.01	0.09 ^a^ ± 0.00	0.09 ^a^ ± 0.00	0.09 ^a^ ± 0.00	0.09 ^a^ ± 0.00
C20:2 n-6	1.19 ^a^ ± 0.00	1.20 ^a^ ± 0.00	1.22 ^a^ ± 0.00	1.23 ^a^ ± 0.00	1.22 ^a^ ± 0.00
C20:3 n-6	0.22 ^a^ ± 0.00	0.23 ^a^ ± 0.00	0.22 ^a^ ± 0.00	0.21 ^a^ ± 0.00	0.21 ^a^ ± 0.00
C20:4 n-6 (AA)	0.36 ^b^ ± 0.00	0.37 ^b^ ± 0.00	0.32 ^a^ ± 0.00	0.32 ^a^ ± 0.00	0.31 ^a^ ± 0.00
C22:2 n-6	0.10 ^a^ ± 0.01	0.09 ^a^ ± 0.02	0.09 ^a^ ± 0.01	0.10 ^a^ ± 0.03	0.09 ^a^ ± 0.00
C18:3 (trans)	0.14 ^a^ ± 0.00	0.14 ^a^ ± 0.00	0.15 ^a^ ± 0.01	0.14 ^a^ ± 0.00	0.14 ^a^ ± 0.00
C18:3 n-3 (ALA)	7.69 ^a^ ± 0.00	7.76 ^a^ ± 0.02	7.91 ^a^ ± 0.02	7.86 ^a^ ± 0.00	7.79 ^a^ ± 0.00
C18:4 n-3	0.18 ^a^ ± 0.00	0.26 ^b^ ± 0.00	0.27 ^c^ ± 0.01	0.26 ^b,c^ ± 0.00	0.26 ^b^ ± 0.00
C20:3 n-3	1.38 ^a^ ± 0.00	1.34 ^a^ ± 0.00	1.35 ^a^ ± 0.00	1.38 ^a^ ±0.00	1.40 ^a^ ± 0.00
C20:5 n-3 (EPA)	2.93 ^b^ ± 0.00	2.92 ^b^ ± 0.01	2.80 ^a^ ± 0.02	2.88 ^a,b^ ± 0.02	2.84 ^a,b^ ± 0.02
C22:6 n-3 (DHA)	4.13 ^c^ ± 0.01	4.23 ^d^ ± 0.01	3.42 ^a^ ± 0.01	3.54 ^b^ ± 0.01	3.52 ^b^ ± 0.01
Non identified FAs	5.37 ± 0.09	5.40 ± 0.06	5.34 ± 0.03	5.52 ± 0.02	5.79 ± 0.02
n-3 g/100g	16.30 ^b^ ± 0.02	16.50 ^b^ ± 0.03	15.75 ^a^ ± 0.05	15.92 ^a^ ± 0.02	15.82 ^a^ ± 0.01
n-6 g/100g	16.01 ^a^ ± 0.00	16.12 ^a^ ± 0.01	16.03 ^a^ ± 0.00	16.07 ^a^ ± 0.05	15.91 ^a^ ± 0.01
n-6: n-3	0.98	0.98	1.02	1.01	1.01

* Data expressed as a g FA/100g fat; SFA: Saturated Fatty Acids; MUFA: Monounsaturated Fatty Acids; PUFA: Polyunsaturated Fatty Acids; ^a^, ^b^, ^c^, ^d^, ^e^—mean values marked by different letters in rows (between cooking methods) differ significantly at *p* ≤ 0.05.

**Table 3:** Docosahexaenoic acid (DHA) and eicosapentaenoic acid (EPA) content (mg fatty acid/100 g ready-to-eat salmon).

Long Chain Polyunsaturated n-3 Fatty Acid	Raw	$\mathrm{Heat}\;\mathrm{Treatment}\;\mathrm{Method}\;\bar{x}\;\pm \;\mathrm{SE}$			
		Sous-Vide Method		Roasting (R_180_, 23 min)	Steaming (SP_100_, 16 min)
		57 °C, 20 min (SV_57_)	63 °C, 80 min (SV_63_)		
EPA	199.05 ^a^ ± 6.93	268.24 ^b^ ± 9.13	344.61 ^c^ ± 11.23	414.91 ^d^ ± 14.56	389.68 ^d^ ± 13.49
DHA	280.27 ^a^ ± 9.76	388.94 ^b^ ± 13.46	421.20 ^b^ ± 14.35	509.39 ^c^ ± 18.27	482.90 ^c^ ± 16.49

^a^, ^b^, ^c^, ^d^—mean values marked by different letters in rows (between cooking methods), differ significantly at *p* ≤ 0.05.
